# Proteome-wide Mapping of Endogenous SUMOylation Sites in Mouse Testis*[Fn FN2]

**DOI:** 10.1074/mcp.M116.062125

**Published:** 2017-03-13

**Authors:** Lili Cai, Jun Tu, Lei Song, Zhihua Gao, Kai Li, Yunzhi Wang, Yang Liu, Fan Zhong, Rui Ge, Jun Qin, Chen Ding, Fuchu He

**Affiliations:** From the ‡State Key Laboratory of Genetic Engineering and Collaborative Innovation Center for Genetics and Development, Institutes of Biomedical Sciences, School of Life Sciences, Fudan University, Shanghai 200032, China;; §State Key Laboratory of Proteomics, Beijing Proteome Research Center, Beijing Institute of Radiation Medicine; National Center for Protein Sciences (The PHOENIX center, Beijing), Beijing 102206, China;; ¶Department of Biochemistry and Molecular Cell Biology, Shanghai Key Laboratory for Tumor Microenvironment and Inflammation, Shanghai Jiao Tong University School of Medicine, Shanghai 200025, China

## Abstract

SUMOylation is a reversible post-translational modification involved in various critical biological processes. To date, there is limited approach for endogenous wild-type SUMO-modified peptides enrichment and SUMOylation sites identification. In this study, we generated a high-affinity SUMO1 antibody to facilitate the enrichment of endogenous SUMO1-modified peptides from Trypsin/Lys-C protease digestion. Following secondary Glu-C protease digestion, we identified 53 high-confidence SUMO1-modified sites from mouse testis by using high-resolution mass spectrometry. Bioinformatics analyses showed that SUMO1-modified proteins were enriched in transcription regulation and DNA repair. Nab1 was validated to be an authentic SUMOylated protein and Lys^479^ was identified to be the major SUMOylation site. The SUMOylation of Nab1 enhanced its interaction with HDAC2 and maintained its inhibitory effect on EGR1 transcriptional activity. Therefore, we provided a novel approach to investigating endogenous SUMOylation sites in tissue samples.

Small ubiquitin-like modifier (SUMO)^1^ is a reversible post-translational protein modifier ubiquitously expressed throughout the eukaryotic kingdom. Mammalian cells express three major SUMO paralogs, namely, SUMO1, SUMO2, and SUMO3. SUMO2 and SUMO3 are ∼95% identical to each other, whereas SUMO2 and SUMO3 are each ∼45% identical to SUMO1. SUMOylation is a covalent, reversible modification that can add one of three SUMO proteins to lysines on target proteins. Similar to ubiquitination, the conjugation of mammalian SUMO to protein substrates requires the E1 activating enzyme (SAE1/SAE2), E2 conjugase (Ubc9), and, in some cases, E3 ligases (1, 2). SUMO proteins can be deconjugated from substrates via the Sentrin-specific proteases (SENPs). Six mammalian SENPs exist, *i.e.* SENP1, SENP2, SENP3, SENP5, SENP6, and SENP7 (3). Protein SUMOylation is associated with many fundamental pathways in both nucleus and cytoplasm including nuclear transport, transcription regulation, DNA replication, DNA repair, genome stability, and cell cycle progression (1, 4, 5).

Ubc9 catalyzes the formation of an isopeptide bond between the C-terminal glycine of SUMOs 1–3 and an ε-amino group of the target lysine by direct interaction with a typical consensus motif ψKxE/D (where ψ is a large hydrophobic amino acid residue and *x* is any residue) present in protein substrates (6, 7). However, many SUMOylation sites remain in nonconsensus motif, such as Lys^164^ of PCNA (8, 9). Therefore, bioinformatics prediction for SUMOylation sites is not sufficiently accurate.

An in-depth understanding of SUMOylation by the direct identification of endogenous SUMO sites at the proteome scale is essential for accessing its physiological and pathological functions. By using proteomic strategies, researchers can identify the global SUMOylation proteome through the purification of SUMOylated targets. However, the low abundance of SUMOylated proteins and dynamic nature of this modification hinder the large-scale identification of protein SUMOylation and mapping of SUMOylated sites by mass spectrometry (MS) in mammalian cells. In addition, after trypsin digestion, mammalian SUMO paralogs remain a relatively long remnant peptide (19 and 32 amino acids, respectively, for mammalian SUMO1 and SUMO2/3), which leads to complex MS/MS fragmentation ion patterns. Consequently, the subsequent MS identification becomes challenging. To this end, great efforts have been made in recent years to develop methods of identifying SUMOylation sites. Previous studies have developed a strategy of overexpressing tagged SUMO plasmids with mutation, such as TGG/RGG, to facilitate the MS identification of SUMO-modified sites. With the aid of affinity purification, tagged SUMO has been successfully used to identify SUMO targets on a global scale (10–24). Vertegaal's group used a similar approach to map SUMO2/3-modified sites (25) and identified over 4300 SUMOylation sites (21). Hay RT's group introduced K-ε-GG antibody into SUMO proteome research and eventually mapped 1002 SUMO2-modified sites (22). Although purification strategies with tagged SUMO have been successfully used to identify SUMO targets on a global scale, this approach is confined to cells and genetically engineered organism applications, thereby providing limited insight into the endogenous regulation of target SUMOylation. In order to get deeper insights into the physiological function of SUMO modification, some researchers have begun to focus on the study of endogenous SUMO modification. Becker *et al.* (26) have developed a protocol that can enable the enrichment of endogenously SUMOylated proteins but cannot identify SUMOylation sites. To date, there are limited approaches that can directly identify endogenous SUMOylation sites. Hendriks *et al.* generated an approach named PRISM (Protease-Reliant Identification of SUMO Modification), which can be successfully used to identify modification sites of wild-type SUMO (27). However, they still studied overexpressed His-tagged SUMO rather than endogenous SUMO, because this approach did not solve the problem of endogenous SUMOylated protein/peptide enrichment. So far, there is still no method for both endogenous wild-type SUMOylated peptides purification and SUMOylation sites identification.

In the present study, we generated a pan-SUMO1 antibody specific to the C-terminal of SUMO1 remnant. Using a dual-high-resolution MS platform, we identified 53 high-confidence endogenous SUMO1-modified sites from mouse testis. The enrichment of modification sequence confirmed the consensus KxE motif observed in previous functional researches. Gene ontology (GO) term analysis revealed that SUMO1-modified proteins were dominantly located in nucleus and were enriched in transcription regulation and DNA repair. We validated the SUMOylation site of Nab1 identified in this study by immunoprecipitation (IP) assay, and revealed that K480R mutant NAB1 impaired its interaction with HDAC2 and attenuated the inhibitory effect of wild-type NAB1 on EGR1 transcriptional activity. Our study provided a strategy for investigating SUMOylation in animal tissues and clinical samples, rendering better understanding of SUMOylation in biological processes.

## EXPERIMENTAL PROCEDURES

### 

#### 

##### Plasmids, Primers, and Antibodies

The coding sequence of mouse Eid3, mouse Nsrp1, mouse Nab1, and human NAB1 were cloned into the vector pRK7-N-Flag for transient transfection. All mutants were generated by site-directed mutagenesis. The expression vectors for SUMO1 were previously constructed and preserved in our laboratory (28). The plasmid HA-HDAC2 was a kind gift from Professor Xiong Yue. All plasmids were confirmed by sequencing before use. Detailed information on the primers and antibodies used is listed in the supplemental Table S4 and S5.

##### Peptide Synthesis and Polyclonal Anti-SUMO1 Antibody Production

The polypeptide C-ELGMEEEDVIEVYQEQTGG-K (the last glycine is attached onto the εNH_2_ of lysine) was synthesized as the antigen. After protein A and antigen-affinity purifications, the polyclonal antibody (TC-Ab) was obtained.

##### Verification of the Affinity of Homemade Antibody

One 90 mm dish of HEK293T cell was transfected with Flag-tagged SUMO1 and Ubc9 plasmids to increase the overall SUMOylation level. After 48 h of transfection, cells were rinsed using PBS, lysed in lysis buffer (0.5% Nonidet P-40, 0.1% SDS, 50 mm Tris-HCl (pH 7.4), 150 mm NaCl, 1 mm EDTA freshly added with PMSF, NEM (N-Ethylmaleimide), and protease inhibitor mixture), sonicated, and centrifuged at 14,000 × *g* for 15 min. The supernatant was transferred to a new tube and protein concentration was determined using a BCA Protein Assay Kit (Beyotime, Nantong, China). Then the lysates were divided into four groups and each group contained 1 mg protein sample. The same amount (0.5 μg) of antiserum, TC-Ab antibody, a commercial SUMO1 antibody, and the control IgG were separately added to the lysates for binding. After 6 h of incubation, protein A/G agarose beads (Merck, Darmstadt, Germany) were added, and the mixture was incubated for another 2 h. The beads were washed three times and boiled for 10 min for sample preparation. Finally, the input and IP samples were subjected to immunoblot analysis and detected using the corresponding primary antibodies.

##### Mouse Tissue Processing and Protein Extraction

Mice were kept on a 12 h light/dark cycle with free access to food and water and housed in accordance with international guidelines. Then, 8 to 10 weeks old C57BL/6 mice were obtained from Slaccas. Excised tissues were shock frozen under liquid nitrogen and stored at −80 °C. Tissue samples were lysed with SUMO lysis buffer (7 M urea, 2 m thiourea, 100 mm Na_2_HPO_4_/NaH_2_PO_4_, 50 mm Tris-HCl (pH 8.0), 1 mm PMSF, 20 mm NEM, and protease inhibitor mixture) using Tissuelyser-24 (Jinxin, Shanghai, China) to grind for 3 min. After continuous rotation for 1 h in 4 °C to promote cracking, the lysate was centrifuged at 16 000g for 15 min, and the supernatant was transferred to a new tube. Protein concentration was determined by Braford assay. For Western blot assay, loading buffer was added to lysate and boiled for 10 min at 95 °C. Before Western blot assay, the sample volume was adjusted by measuring the gray-scale value using Coomassie brilliant blue staining. The protein samples from different tissues were subjected to Western blot and detected by anti-SUMO1 antibody (Abcam). For MS sample preparation, lysate was then supplemented with 10 mm dithiothreitol (DTT) and incubated at 56 °C for 30 min for protein reduction. After cooling to room temperature, 50 mm iodoacetamide (IAA) was further added to the lysate and incubated for another 30 min at room temperature. The proteins were then precipitated by prechilled acetone supplementation at −20 °C. Protein pellet was collected by centrifugation at 800g for 10 min. Acetone was carefully removed, and the proteins were vacuum dried.

##### Sample Preparation and Peptide IP

Proteins were resuspended with 50 mm NH_4_HCO_3_ (pH 8.0) (for Trypsin digestion) or 20 mm Tris-HCl (pH 9.0–9.5) (for Lys-C digestion) and subjected to short sonication. Trypsin (Sigma, St Louis, MO) or Lys-C (Wako, Osaka, Japan) was added to the sample at enzyme-to-protein ratio of 1:75 and incubated at 37 °C overnight. Additional enzyme was added at 1:150 ratio for another 3 h of digestion. After Lys-C digestion, HCl was added to adjust the pH to 7.4. The supernatant was vacuum dried. We have made a cross-linking to fix the homemade antibody to agarose beads (Thermo Fisher Scientific, San Jose, CA) matrix or Dynabeads (Invitrogen, Carlsbad, CA) before performing IP assay. The antibody cross-linking procedure was performed according to the manufacturer's instructions. Briefly, cyanoborohydride solution was used as the cross-linking reagent for agarose beads. For Dynabeads^®^ M-280 Tosylactivated, any ligand containing amino or sulfhydryl groups can be covalently coupled to the bead surface, and ammonium sulfate is used for the antibody coupling to the beads. Peptides were redissolved using 0.5% NETN buffer (0.5% Nonidet P-40, 50 mm Tris-HCl (pH 7.4), 150 mm NaCl, 1 mm EDTA, and protease inhibitor mixture) and cross-linked TC-Ab antibody (150 μl Dynabeads or 100 μl agarose beads) was added to the peptide mixtures and incubated at 4 °C overnight while rotating. The peptides were eluted three times from the beads with 200 μl of elution buffer (0.1% formic acid and 5% acetonitrile). The supernatant was vacuum dried. Peptides were redissolved using 25 mm NH_4_HCO_3_, and 2 μg of Glu-C (Roche, Basel, Switzerland) was added to the sample for digestion at 25 °C overnight. Additional Glu-C (1 μg) was added for another 4 h of digestion. The supernatant was vacuum dried for LC-MS/MS analysis.

##### Immunoblot Analysis

Protein samples were boiled for 10 min in Laemmli buffer (10% (w/v) glycerol, 2% SDS, 10% (v/v) β-mercaptoethanol, and 62.5 mm Tris-HCl (pH 6.8)) and separated by SDS-PAGE before transferring onto nitrocellulose membrane. After blocking with 5% non-fat milk, the membranes were incubated with primary antibodies (SUMO1, Flag, or HA), followed by a horseradish peroxidase-conjugated secondary antibody. The secondary antibody was detected with an ECL chemiluminescence detection system (GE Healthcare, Stockholm, Sweden).

##### Cell Culture and SUMOylation Assay

HEK293T cells were cultured in DMEM medium (Gibco, Grand Island, NY) containing 10% fetal bovine serum (Gibco) at 37 °C in 5% CO_2_ constant atmosphere. After 48 h of transfection, cells were rinsed using PBS, lysed in lysis buffer (0.5% Nonidet P-40, 0.1% SDS, 50 mm Tris-HCl (pH 7.4), 150 mm NaCl, 1 mm EDTA freshly added with PMSF, NEM, and protease inhibitor mixture), sonicated, and centrifuged at 14,000 × *g*. Then anti-Flag M2 beads were added to the supernatant and incubated overnight at 4 °C. The total amount of protein lysates was determined by BCA protein assay. Beads were washed three times with 0.25% NETN buffer (0.25% Nonidet P-40, 50 mm Tris-HCl (pH 7.4), 150 mm NaCl, and 1 mm EDTA), and further boiled for sample preparation. Then, the samples were subjected to further SDS-PAGE and immunoblot analysis.

##### Luciferase Reporter Assays

The genomic DNA fragment of the promoter region and 5′-upstream regulatory sequence of *EP300* (29) was amplified and cloned into pGL3-Basic vector (Promega, Madison, WI). The constructed plasmids were verified by sequencing. HepG2 cells were transfected with different plasmids combinations, and pRL-TK renilla luciferase reporter plasmid was used as an internal control. Cells were lysed 36 h after transfection, and luciferase activities were assessed using Dual-Luciferase Reporter Assay System (Promega). All data were measured on a Modulus Microplate Multimode Reader (Turner Biosystems, Sunnyvale, CA).

##### MS Analysis

LC-MS/MS analyses were performed on an Easy-nLC 1000 liquid-chromatography system (Thermo Fisher Scientific) coupled with a Q-Exactive Plus/Q-Exactive HF through a nano-electrospray ion source (Thermo Fisher Scientific).

For Q-Exactive Plus, The peptide mixture was eluted from a 360-μm ID × 2 cm, C18 trap column and separated on a homemade 100 μm ID × 10 cm column (C18, 1.9 μm, 120 Å, Dr. Maisch GmbH) with a linear 5–35% acetonitrile gradient at 500 nL/min. Survey scans were acquired after accumulating of 3e6 ions in Orbitrap for *m*/*z* 300–1400 using a resolution of 70,000 at *m*/*z* 400. The top 20 intense precursor ions were selected for fragmentation in the HCD cell at a normalized collision energy of 27%, and then fragment ions were transferred into the Orbitrap analyzer operating at a resolution of 17,500 at *m*/*z* 400. The dynamic exclusion of previously acquired precursor ions was enabled at 18 s.

For Q-Exactive HF, the peptide mixture was eluted from a 360-μm ID × 2 cm, C18 trap column and separated on a homemade 150 μm ID × 12 cm column (C18, 1.9 μm, 120 Å, Dr. Maisch GmbH) with a linear 5–35% acetonitrile gradient at 600 nL/min. Survey scans were acquired after accumulating of 3e6 ions in Orbitrap for *m*/*z* 300–1400 using a resolution of 120,000 at *m*/*z* 200. The top 20 intense precursor ions were selected for fragmentation in the HCD cell at a normalized collision energy of 27%, and then fragment ions were transferred into the Orbitrap analyzer operating at a resolution of 15,000 at *m*/*z* 200. The dynamic exclusion of previously acquired precursor ions was enabled at 18 s.

##### Data Processing

Raw MS data files were analyzed by MaxQuant (version 1.5.3.30) (30). The first search was carried out with a mass accuracy of 20 ppm, whereas the main search used 4.5 ppm for precursor ions. Mass tolerance of MS/MS spectra was set to 20 ppm to search against mouse protein RefSeq database (released 1 July 2013, 27414 proteins). Database searches were performed with Trypsin/P (K/R) (or Lys-C/P (K)) and Glu-C (E) specificity and four missed cleavage sites were allowed. Peptides were accepted with a minimum length of 7 amino acids and a maximum size of 4.6 kDa, Acetyl (Protein-N term), oxidation (M), carbamidomethylation (C), NEM (C), SUMOylation (K_QTGG, monoisotopic 343.14918, not in C-term) were chosen as variable modifications. For some experiments (EndoSUMO1–01/02/04/05/07/09/10/12), because NEM was not used in the initial procedure, carbamidomethylation (C) was considered as a fixed modification and three others as variable modifications. The processed data was filtered by posterior error probability (PEP) to achieve a protein false discovery rate (FDR) of below 1%, a peptide-spectrum match FDR of below 1%, and in addition a site decoy fraction of 1% was set (30). SUMO-site peptides were additionally filtered to have an Andromeda score of at least 40, a localization score of at least 40, a localization probability of at least 90%.

##### Bioinformatics Analysis

Sequence analysis was performed with Motif-X (31, 32). All the peptides for high-confidence SUMO1 sites were pre-aligned and used for the analysis, and 13 amino acid sequences were chosen for input. GO enrichment analysis was performed by DAVID (version 6.8) (33, 34). Network analysis was performed by Ingenuity Pathway Analysis (IPA, Ingenuity Systems, Redwood City, CA).

##### Experimental Design and Statistical Rationale

**Fig. 1.**
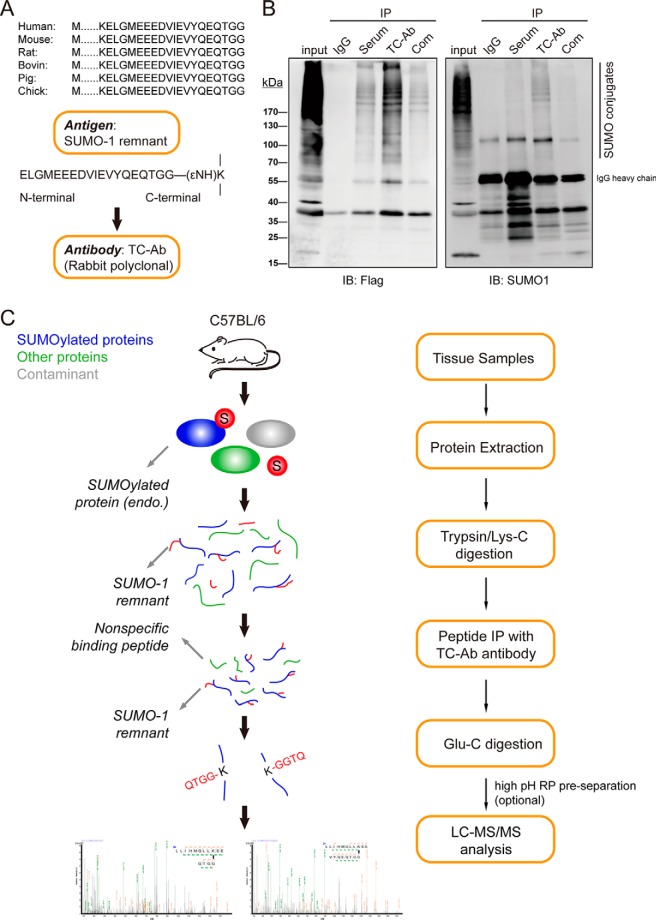
**Strategy for mapping endogenous SUMO1-modified sites.** *A*, Sequence alignment of SUMO1 orthologs and strategy of antibody preparation. The tryptic remnant peptide of SUMO1 C-terminal attached to εNH_2_ of lysine was synthesized as an antigen to immunize rabbits and obtain a polyclonal antibody specifically identifying SUMO1-modified peptide. *B*, Verification of the efficiency of TC-Ab antibody. HEK293T cells were transfected with Flag-SUMO1 and Ubc9-Myc plasmids to enhance the overall SUMOylation level. Whole cell lysates were divided into groups for IP assay. The same amount of antiserum, TC-Ab antibody, and commercial SUMO1 antibody were used to enrich SUMOylated proteins. IgG was used as the negative control. Com refers to a commercial SUMO1 antibody. *C*, Schematic overview of endogenous SUMO1-modified peptides identification. Tissue samples were subjected to trypsin/Lys-C protease digestion, followed by peptide IP to enrich SUMO1-modified peptides. After Glu-C protease digestion, QTGG featured peptides were successfully exposed for further MS identification. High pH RP pre-separation is optional before LC-MS/MS.

A homemade SUMO1 tryptic remnant antibody was prepared by using the synthesized ELGMEEEDVIEVYQEQTGG-(εNH)K peptide. This antibody was then used for the identification of endogenous SUMO1 modification sites of mouse testis tissue. As shown in Fig. 1*C*, mouse tissue samples were subjected to proteolytic digestion (Trypsin or Lys-C protease). The antibody was incubated with peptide mixture to enrich SUMO1-modified peptides. As the remnant SUMO1 chain is too long to be identified by MS, we executed the second enzyme digestion with Glu-C to obtain a much shorter remnant SUMO1 side chain (QTGG), thus facilitating MS identification for endogenous SUMO1-modified sites.

In each experiment (EndoSUMO1–45 was a special case that would be mentioned in detail), four testis tissues from two mice were lysed as one sample. The initial amount of protein sample was 40–50 mg. Twenty-six experiments (biological replicates) and one control experiment were performed. Detailed information of each experiment was explained in supplemental Table S6. We got 37 raw files in total, including 2 Lys-C raw files and 35 Trypsin raw files (one control raw file included). All the raw files were searched using MaxQuant.

Statistical analysis method used in this study was Student's *t* test.

## RESULTS

### 

#### 

##### Generation of Antibody Targeting SUMO1-modified Peptides and Design of Streamlined MS Platform for Identifying Endogenous SUMO1 Modification Sites

For ubiquitination, commercial K-ε-GG antibody can be used to enrich di-glycine modified peptides (35–37). However, for SUMO1-modified peptide purification, antibodies are not commercially available. To generate SUMO1 antibody, we aligned the SUMO1 sequence of different species. The tryptic remnant“ELGMEEEDVIEVYQEQTGG” in the very C-terminal of SUMO1 is highly conserved cross species (Fig. 1*A*). We then synthesized ELGMEEEDVIEVYQEQTGG-(εNH)K as antigen to prepare a polyclonal antibody. The homemade SUMO1 tryptic remnant antibody (*i.e.* TC-Ab antibody) showed higher affinity than commercialized SUMO1 antibody (Fig. 1*B*) and could specifically recognize SUMO1 rather than SUMO2/3 (supplemental Fig. S1). To identify endogenous SUMO1 modification sites, we designed a streamlined pipeline. Briefly, proteins were extracted from cells or tissues. Trypsin or Lys-C protease was used for proteome digestion to release SUMO1-modified peptides with ELGMEEEDVIEVYQEQTGG-(εNH)K remnant. TC-Ab antibody was incubated with peptide mixture to enrich SUMO1-modified peptides. Given that the remnant SUMO1 chain was too long to be identified by MS, we used a second enzyme, Glu-C protease, to trim the trypsin remnant SUMO1 side chain to QTGG, thereby facilitating MS identification. The digestion product was then submitted to dual-high-resolution MS for identification (Fig. 1*C*).

##### SUMO1 Modification Was Dominantly Activated in Mouse Testis

**Fig. 2.**
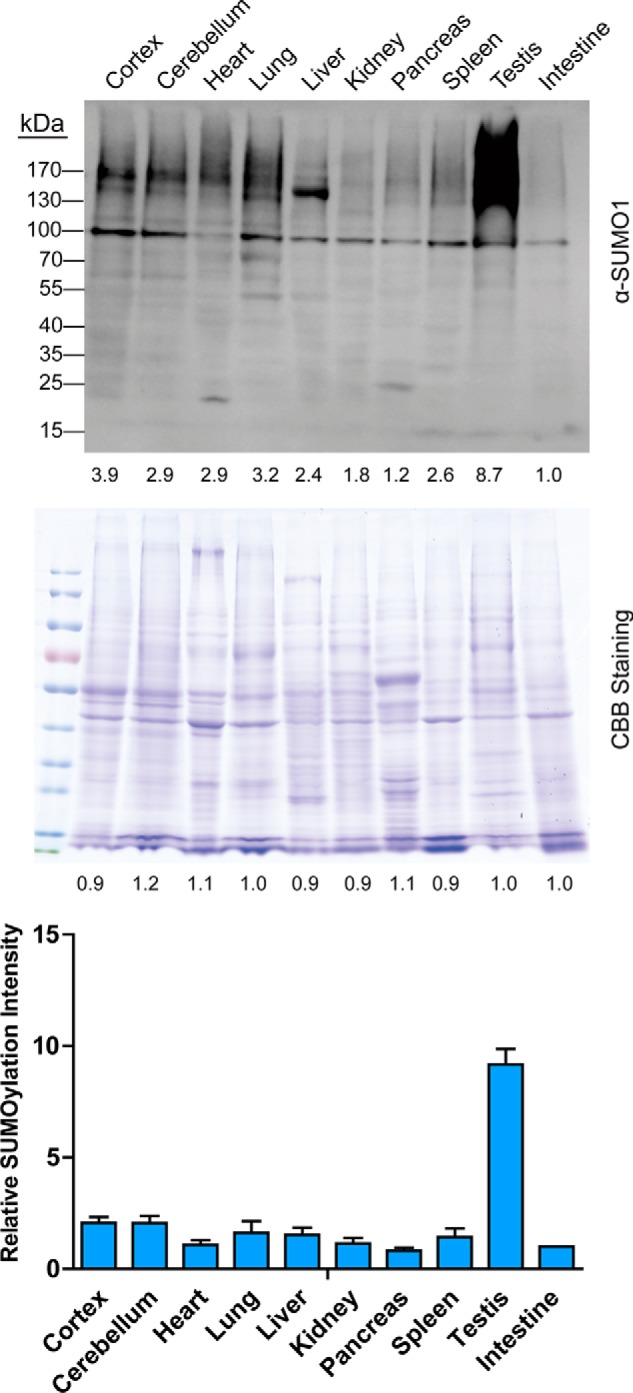
**Mouse testis is highly SUMOylated *in vivo*.** SUMO1-modified proteins are highly enriched in mouse testis. The excised tissues from C57BL/6 mice were lysed and treated for immunoblot using SUMO1 antibody (Abcam) to detect the SUMOylation status of different tissues (up). Coomassie brilliant blue staining indicates the same amount of loading of each lane (middle). The bottom figure shows the comparison of the gray-scale value of SUMOylated bands (bottom). Results are displayed as mean ± S.E. from quadruplicate experiments of four mice.

We used SUMO1 antibody to survey the basal SUMO1 modification level of mouse tissues. As shown in Fig. 2, 10 tissues were accessed including cortex, heart, lung, liver, testis, and so on, in which testis represented the highest endogenous basal SUMOylation level among all other tissues. Furthermore, we analyzed the mRNA level of SUMOylation-related enzymes and found that SUMO-activating enzyme (Uba2), SUMO-conjugating enzyme (Ubc9), and E3 SUMO-protein ligases (Pias2, Pias4) showed higher expression in mouse testis (supplemental Fig. S2). These results indicated that SUMOylation bioprocess was activated in the testis, revealing an active role of SUMO in the testis.

##### Identification of Endogenous SUMO1 Modification Sites in Mouse Testis

We next used the above SUMO1 immunoaffinity profiling approach to identify endogenous SUMO1 modification sites in mouse testis. Twenty-six independent experiments were performed and 36 raw files were obtained in total. The MaxQuant search engine was applied for database search, and the SUMO1-modified sites that were at least assigned by two spectra were defined as high-confidence sites.

**Fig. 3.**
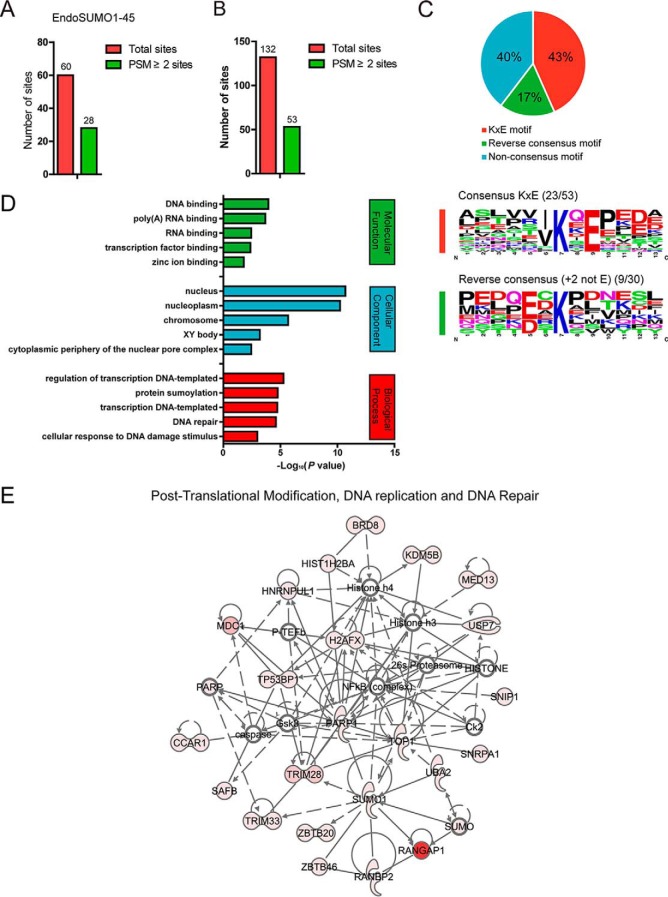
**Identification and bioinformatics analysis of endogenous SUMO1-modified proteins and sites.** *A* and *B*, Bar chart shows endogenous SUMO1-modified sites identified in one experiment (sample EndoSUMO1–45) (*A*) and all experiments in this study (*B*). The red bar represents the number of total putative SUMO1-modified sites and the green bar represents the number of high-confidence sites which were assigned at least by two spectra. *C*, SUMO1-modified lysines are enriched in the KxE consensus motif. The pie chart shows distribution of identified endogenous SUMO1 modification sites based on sequence motif (upper). SUMO1 sites located within consensus KxE sequence motif are represented in red, sites located at a reverse consensus motif are represented in green. The bottom panel shows the graphical representation of SUMO site motifs identified in this study. *D*, GO enrichment analysis of potential endogenous SUMO1-modified proteins. The bar plot shows the top 5 terms of significantly enriched biological process, cellular component and molecular function for potential SUMO targets. All the analysis results were listed in supplemental Table S7. *E*, Network analysis of identified endogenous SUMO1-modified proteins. Network enrichment representation of SUMOylated proteins associated with Post-Translational Modification, DNA Replication, and Repair.

For one of the experiments (sample EndoSUMO1–45), the initial amount of proteins was doubled. After peptide IP and Glu-C digestion, we performed a high pH reverse-phase chromatography separation to divide the sample into three fractions and finally got nine MS raw files. We have identified 60 putative SUMO1-modified sites through MaxQuant search. Twenty-eight sites were at least assigned by two spectra, which further confirmed the feasibility of our approach (Fig. 3*A* and supplemental Table S1).

Combining all the results from 36 raw files, 132 putative SUMO1-modified sites were identified in mouse testis by MaxQuant, of which 53 sites on 37 proteins were defined as high-confidence sites (PSM ≥ 2) (Fig. 3*B* and supplemental Table S2). In addition, 30 SUMOylation sites in this study were also identified by other studies with the homologous sequences of human (21, 23), such as Rangap1 (Lys^526^), Top1 (Lys^119^), Mdc1 (Lys^1462^), Safb (Lys^316^), Trim28 (Lys^779^), and Zmym4 (Lys^250^). As expected, Rangap1 was the most abundant SUMO1-modified protein under physiological conditions, which was consistent with the previous studies (26, 38). Moreover, we also identified some novel targets, such as Sox30 (Lys^242^ and Lys^336^) and Eid3 (Lys^29^) for the first time in mouse tissue.

In addition, we also evaluated all the raw files using another two search engines, Mascot and pLink (39), to identify endogenous SUMO1-modified peptides. For all the raw files, 51 putative SUMO1 sites (33 of which were identified at least twice) were identified by Mascot, and 116 putative SUMO1 sites (44 of which were identified at least twice) were identified by pLink (supplemental Fig. S3*A*, and supplemental Table S3). Among 53 high-confidence SUMO1 sites identified by MaxQuant, 25 sites were also identified by Mascot or pLink, indicating the bias of different search engines for SUMOylation sites mapping (supplemental Fig. S3*B*, supplemental Table S2 and S3). Several sites identified only by Mascot or pLink were also assigned by multiple spectra, such as Top1-Lys^155^, Mdc1-Lys^1497^, Ranbp2-Lys^2411^, and Nsrp1-Lys^209^.

##### Features of Endogenous SUMO1-modified Proteins

We used the 53 high-confidence SUMO1-modified sites for the further bioinformatics analysis and found that 43.4% of identified sites (23 sites) were located within the consensus KxE motif (Fig. 3*C*). Among all KxE sites, 21 sites were located at the most consensus I/V/LKxE motif. In addition, nine of the rest sites (17%) were located within the reverse E/DxK motif (Fig. 3*C*) (25). We then provided visual sequence logos of KxE motif and the reverse consensus motif for these SUMO1-modified sites (Fig. 3*C*). The high percentage of KxE motif further confirmed the quality of our endogenous SUMO1-modified sites.

GO term analyses revealed a significant enrichment of identified high-confidence proteins in the nucleus (*p* = 2.03E-11) (Fig. 3*D*, and supplemental Table S7), which were consistent with previous studies in SUMO field. These proteins were enriched in the functional groups associated with transcription regulation (*p* = 5.40E-06) and DNA repair (*p* = 2.51E-05) (Fig. 3*D*, and supplemental Table S7). We then submitted our SUMO1 modified protein list to Ingenuity Pathway Analysis (IPA) to establish protein-interaction networks. Results also showed that the SUMO1-modified proteins were enriched in the network of Post-Translational Modification, DNA Replication, and Repair (score = 61) (Fig. 3*E*).

##### Validation of Endogenous SUMO1 Targets of Mouse Testis

To further validate the endogenous SUMO1 target proteins identified in our screens and their associated SUMO1-modified sites, we selected Nab1 (NGFI-A binding protein 1) and Eid3 (EP300-interacting inhibitor of differentiation 3) for subsequent *in vivo* SUMOylation assay and site-directed mutagenesis experiments. Nab1 belongs to the NAB family and acts as a transcriptional repressor for zinc finger transcription factors EGR1 and EGR2 (40). Eid3 is highly expressed in testis and may acts as a potent repressor of nuclear receptor-dependent transcription (41). Lys^479^ of Nab1 and Lys^29^ of Eid3 were identified as SUMO1-modified sites in our study (supplemental Fig. S4, supplemental Table S1 and supplemental Table S2).

**Fig. 4.**
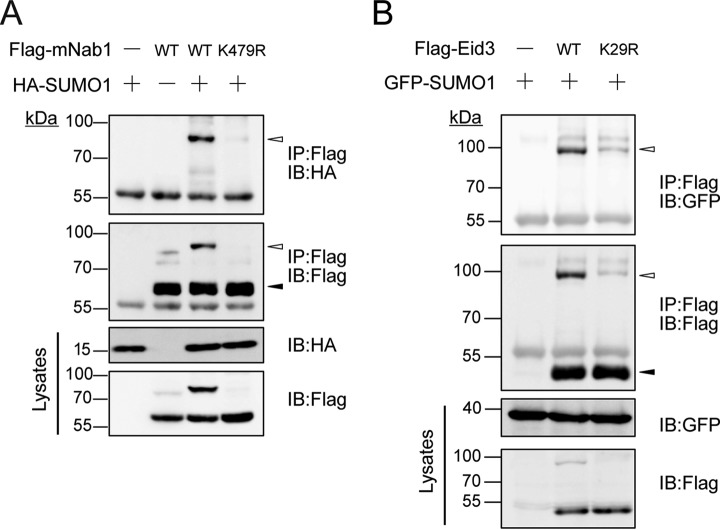
**Validation of endogenous SUMO1-modified targets of mouse testis.** *A* and *B*, HEK293T cells were cotransfected with HA/GFP-tagged SUMO1 and Flag-tagged wild-type or K-to-R mutant expression plasmids for selected candidates, that is, Nab1 (*A*) and Eid3 (*B*). Cell lysates were subjected to IP with anti-Flag M2 beads, and SUMOylated bands were detected by Western blot analysis using anti-Flag and anti-HA (or anti-GFP) antibodies. Black arrows represent unmodified candidate proteins, and the white represent the SUMO1-modified forms.

We then constructed Flag-tagged Nab1 and Eid3 expression plasmids and the corresponding K-to-R mutants. The *in vivo* SUMOylation assay was performed to detect the protein SUMOylation through immunoblot. HEK293T cells were cotransfected with HA/GFP-tagged SUMO1 vector and plasmids encoding the different Flag-tagged candidates or the corresponding mutants. After cell lysis, SUMOylated proteins were purified using anti-Flag M2 beads, and the SUMO1-modified forms of the candidates were detected through immunoblot. Western blot results confirmed that Lys^479^ of Nab1 and Lys^29^ of Eid3 identified in our study were bona fide SUMO1-modified sites (Fig. 4*A* and 4*B*).

In addition, we also chose Nsrp1 (Nuclear speckle splicing regulatory protein 1, synonym: Ccdc55, Nsrp70), which was exclusively identified by pLink search engine, as another candidate for validation (supplemental Table S3). Nsrp1 is a RNA-binding protein that mediates pre-mRNA alternative splicing regulation (42). Lys^210^ of human homolog NSRP1 was previously identified as a SUMO2-modified site by MS (21). In our study, we identified Lys^209^ of mouse Nsrp1 as an endogenous SUMO1-modified site from pLink searching. Through IP-Western blot, we also validated Nsrp1 as a SUMO1 target and confirmed Lys^209^ as its SUMO1 acceptor site (supplemental Fig. S5*A*).

##### SUMOylation Maintained the Inhibitory Effect of NAB1 Toward EGR1 Transcriptional Activity

Human NAB1 protein is reportedly a SUMOylated protein before (43), but the SUMOylation site and function of NAB1 is still unknown. Thus, we chose Nab1 for the further functional study of SUMOylation. We further constructed Flag-tagged NAB1 plasmid from human coding sequence and the corresponding K480R mutant. Through *in vivo* SUMOylation assay, we also confirmed that Lys^480^ was the major SUMO1-modified site of the human NAB1 (supplemental Fig. S5*B*).

**Fig. 5.**
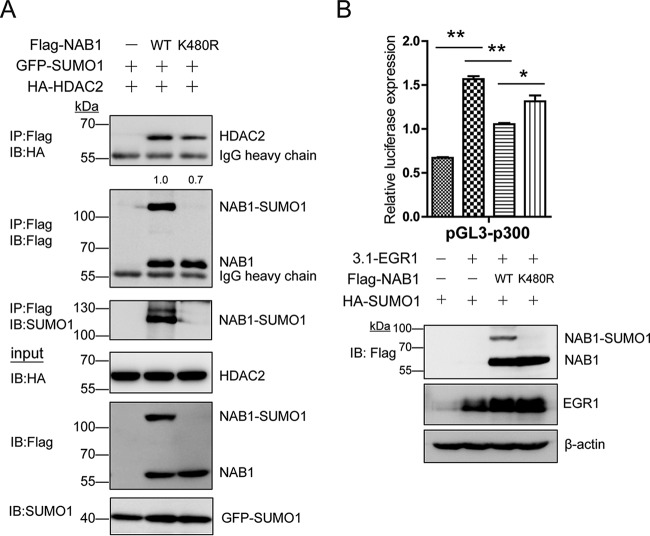
**SUMOylation maintained the transcriptional repressing activity of NAB1.** *A*, Interaction of wild-type and K480R NAB1 with HDAC2. Flag-tagged NAB1 (WT or K480R) was immunoprecipitated with anti-Flag M2 beads. NAB1 and HDAC2 were detected by Western blot analysis using anti-Flag or anti-HA antibodies, respectively. *B*, SUMOylation site mutation impaired the inhibitory effect of NAB1 on EGR1 transcriptional activity. Luciferase reporter assay used *EP300* promoter as reporter construct and co-transfected it with different combination of wild-type or mutant NAB1 plasmids in HepG2 cells with EGR1 and SUMO1 plasmids. Data were corrected for *Renilla* activity. * *p* < 0.05, ** *p* < 0.01, unpaired Student's *t* test.

NAB corepressors (NAB1 and NAB2) play a critical role in the regulation of EGR transcriptional activity. NAB2 reportedly represses transcription by interacting with the CHD4 subunit of the nucleosome remodeling and deacetylase (NuRD) complex (44). Considering that NAB1 and NAB2 share a high degree of homology (45), NAB1 might also presumably be able to interact with one of the components of corepressor complex such as histone deacetylase (HDAC). As previously reported, SUMO-modified Elk-1 can recruit HDAC2 as a corepressor to responsive promoters, thereby representing a repressive function (46). Thus, we hypothesized that SUMOylation of NAB1 may recruit HDAC2 for conducting repression activity. Accordingly, we determined whether SUMOylation of NAB1 affected its binding ability with HDAC2. Flag-NAB1 and HA-HDAC2 were co-transfected in HEK293T cells, and IP assay was performed using anti-Flag M2 beads. As the results show, NAB1 can interact with HDAC2, and wild-type NAB1 co-immunoprecipitated more efficiently with HDAC2 than with K480R mutant (Fig. 5*A*). Our results suggested that the SUMO1 modification of NAB1 promoted the recruitment of HDAC2 in NuRD complex which might mediate the inhibitory effect of Nab on the transcriptional activity of EGR1.

We then determined whether the Lys^480^ SUMOylation of NAB1 was involved in repressing EGR1 transcriptional activity. *EP300* was previously reported as a target gene of EGR1 in prostate cells (29). We then constructed the reporter gene plasmid of *EP300* promoter region. The dual-luciferase reporter assay was performed to detecting the relative transcriptional activity. We conformed that EGR1 can regulate the promoter region of *EP300* in HepG2 cells (supplemental Fig. S6). As expected, wild-type NAB1 repressed the transcriptional activity of EGR1. However, K480R mutant NAB1 significantly reduced the inhibitory effect (Fig. 5*B*), demonstrating that the Lys^480^ SUMOylation of NAB1 played an important role in repressing EGR1 transcriptional activity. Thus, our data suggested that the SUMOylation of NAB1 recruited HDAC2 to repress the transcriptional activity of EGR1.

## DISCUSSION

Several studies have made great effort in the enrichment of endogenously SUMOylated proteins (26, 47), but few methods can directly map endogenous SUMO acceptor sites on a global scale yet. In the present work, we provided a method that enabled the enrichment of endogenous SUMO1-modified peptides and directly mapped endogenous SUMO1 acceptor sites in mouse tissue for the first time. We applied such a method to map the endogenous SUMO1 modification sites of proteins, resulting in the identification of 53 high-confidence SUMO1-modified sites on 37 endogenous proteins in mouse testis.

The enrichment of modified peptides/proteins is a key step of post-translational modification study, and the enrichment of peptides has more advantages than proteins. To date, no commercial antibody can be used to purify SUMO1-modified peptides. In the present study, we provided a high-affinity SUMO1 antibody for SUMO1-modified peptides enrichment. Our approach not only solved the enrichment step of endogenous SUMO1-modified peptides but also gave the solution for directly identifying endogenous SUMO1-modified sites. More importantly, the application of our approach can also be extended to animal tissues and clinical samples that are unavailable for analysis before. In addition, if we want to identify endogenous SUMO2/3-modified sites of tissues, we can generate a SUMO2/3-remnant antibody (tryptic remnant peptide for SUMO2/3 is “FDGQPINETDTPAQLEMEDEDTIDVFQQQTGG”) for purification of the endogenous SUMO2/3-modified peptides. After purification, another Asp-C digestion can be applied to trim the peptide into “VFQQQTGG”, and this short peptide can be used as a linker chain for pLink search.

Only a few suitable data-search strategies for SUMOylation analysis exist. Pedrioli *et al.* introduced an automated pattern recognition tool named SUMmOn to detect diagnostic SUMO fragment ion series within complex MS/MS spectra (48). This kind of software was developed to interpret MS2 spectra of branched SUMOylated peptides. In addition, Hsiao *et al.* developed a database search tool (“ChopNSpice”) that can successfully allow the identification of SUMO acceptor sites from endogenous SUMOylated proteins (49). Nevertheless, the application of these two search strategies is not widely used in proteomic analyses of SUMOylation. pLink is a software dedicated for the analysis of chemically cross-linked proteins or protein complexes using MS (39, 50). Protein SUMOylation can be considered as a special case of chemical cross-linking of two proteins, and pLink software can be used to identify SUMOylation sites. The latest version of pLink has been expanded to identify endogenous protein cross-linking sites such as disulfide bonds (51) (pLink-SS) and SUMO modification sites (pLink-SUMO). Therefore, in our study, we also tried pLink software for endo-SUMO1 site identification. Considering the missed cleavage of Glu-C protease on SUMO1 branch chain, VYQEQTGG was used as another linker chain for pLink search apart from QTGG. A total of 44 high-confidence endogenous SUMO1-modified sites were identified by pLink search (supplemental Fig. S3*A*). Moreover, a lot of these sites were also identified by Mascot or MaxQuant, further confirming the feasibility of pLink software for the identification of SUMOylation sites. In addition, we identified Lys^209^ of Nsrp1 as an endogenous SUMO1-modified site only in pLink search and validated its SUMOylation using IP-WB assay (Supplemental Fig. S5*A*), indicating the bias for different search engines. Thus, a more compatible search engine needs to be developed for endogenous SUMOylation identification.

SUMO modification has been proven to play an important regulatory role in various cellular functions. A previous study has suggested that SUMOylation is involved in the regulation of spermatogenesis (52). In the database of the Human Protein Atlas, the distribution of SUMO1/2/3 is specific and complementary within spermatogonium, spermatocyte, and sperm cell (53) (supplemental Fig. S7). In the present study, we confirmed the endogenous SUMO1 modification sites for both Nab1 and Eid3 by IP assays (Fig. 4). Eid3 is specifically expressed in testis, indicating that Eid3 might have testis-specific functions (41). In this study, we mapped Lys^29^ as the major SUMO1-modified site for Eid3, suggesting that SUMOylation of Eid3 might play a role in the regulation of testis functions. Furthermore, we found that SUMOylation of Nab1 plays an important role in transcriptional repression. However, the detail function of SUMOylation in testis still needs further studies. Nevertheless, we provided the endogenous SUMO1 modification profile in mouse testis which facilitated for studying the function of SUMO1 in testis in future.

As we know, meiosis is the most important bioprocess in the testis. DNA damage repair plays an important role in this process. Several important proteins involved in DNA repair pathway such as Mdc1, Trp53bp1 and H2ax were identified in our study. The role of SUMOylation in response to DNA damage has been extensively studied over the past decade. Lys^1840^ of MDC1 has been reported as a SUMO modification site, and the SUMOylation of MDC1 regulated its stability following DNA damage (54). H2AX is also reported to be a SUMO target and is involved in DNA damage response (55). H2AX is specifically accumulated over the X and Y chromosomes in spermatocytes during meiotic sex chromosome inactivation (MSCI) (56), suggesting that SUMOylation might be involved in this process. For Mdc1, we have mapped several new sites in it, indicating that Mdc1 might be a relatively extensively SUMOylated target in mouse testis, and the related work is worthy of further study.

Previous studies on individual proteins suggested that SUMOylation primarily occurs on proteins containing the consensus motif ψKxE/D. Our study confirmed the enrichment of SUMO1-modified sites on the KxE consensus motif with the endogenous SUMOylation proteomics data set for the first time, indicating that SUMOylation sites truly tend to be located within hydrophobic residues and acidic residues. In addition to structural features, we demonstrated that endogenous SUMO1 modification was enriched in nucleus and the functional processes of transcription regulation and DNA repair. We confirmed the endogenous SUMO1 modification site of a transcription repressor Nab1 by IP assay, and found that the SUMOylation of NAB1 may enhance its binding with HDAC2 to maintain its repression for the transcriptional activity of EGR1. This is only a part of the mechanism about how NAB1 represses the transcriptional activity of EGR1, and the underlying mechanism needs further study.

Through our immunoaffinity approach, we directly mapped the endogenous SUMO1-modified sites in mouse testis for the first time. We failed to detect any endogenous SUMO site in the IgG control experiments, revealing the advantage and specificity of our approach in identifying SUMO site. However, there was still a large amount of the nonspecific binding for our antibody, because SUMO1-modified peptides represented a small fraction of the total identified peptides (∼0.5%). In our experiments, we also made a cross-linking to fix the TC-Ab antibody to agarose beads (Thermo Fisher Scientific) or Dynabeads (Invitrogen) and found that Dynabeads generated less nonspecific bindings, resulting in an increase of final peptide purity (from 0.1% (agarose beads) to around 0.5% (Dynabeads)). Nevertheless, the affinity and specificity of the antibody still needs to be optimized. Maybe in the future a better antibody can be prepared and more SUMOylated sites will be identified in physiological and pathological samples using immunoaffinity approach. Meanwhile, we would like to further improve the SUMO site identification coverage and reproducibility.

Taken together, we developed a streamlined strategy that allowed the screening of endogenous SUMO1 conjugation sites in cells and tissues at the proteome scale. In combination with high-resolution MS platform, our strategy can be used to assess functions and regulations of SUMOylation in physiological and pathological bioprocesses.

## DATA AVAILABILITY

The proteomics data reported in this paper have been deposited to iProx database (URL: http://www.iprox.org/) and are available under accession number IPX0000841000.

## Supplementary Material

Supplemental Data

## Footnotes

^1^ The abbreviations used are:

SUMO: Small Ubiquitin-like Modifer
EGR1: Early growth response protein 1
Eid3: EP300-interacting inhibitor of differentiation 3
GO: Gene Ontology
HDAC: Histone deacetylase
IAA: Iodoacetamide
IP: immunoprecipitation
MDC1: Mediator of DNA-damage checkpoint 1
NAB1: NGFI-A binding protein 1
NEM: N-Ethylmaleimide
NSRP1: Nuclear speckle splicing regulatory protein 1
SENP: Sentrin-specific proteases.
