# Supplementary material for: Proteome-wide Mapping of Endogenous SUMOylation Sites in Mouse Testis
Source: Mol Cell Proteomics. 2017 Mar 13;16(5):717–27. doi: 10.1074/mcp.M116.062125 (PMC5417816; doi:10.1074/mcp.M116.062125)
Supplement: Supplemental Data [file supp_16_5_717__index.html]

Proteome-wide mapping of endogenous SUMOylation sites in mouse testis — Proteome-wide Mapping of Endogenous SUMOylation Sites in Mouse Testis — Endogenous SUMOylation of Mouse Testis — Supplemental Data 

# Proteome-wide Mapping of Endogenous SUMOylation Sites in Mouse Testis

## Supplemental Data

- supplement\_text (.pdf, 1.3 MB) - supplemental figures and supplemental table S4-S6
- Supplemental table S1 (.xlsx, 27 KB) - Identified proteins with endogenous SUMO1-modified sites of sample EndoSUMO1-45.
- Supplemental table S2 (.xlsx, 47 KB) - Identified proteins with endogenous SUMO1-modified sites from mouse testis.
- Supplemental table S3 (.xlsx, 158 KB) - List of SUMO1-modified sites identified by Mascot and pLink search engines.
- Supplemental table S7 (.xlsx, 20 KB) - The GO term analysis result using DAVID database
