# Supplementary material for: Proteome-wide Mapping of Endogenous SUMOylation Sites in Mouse Testis
Source: Mol Cell Proteomics. 2017 Mar 13;16(5):717–27. doi: 10.1074/mcp.M116.062125 (PMC5417816; doi:10.1074/mcp.M116.062125)
Supplement: Supplemental Data [file 10.1074_M116.062125_mcp.M116.062125-1.pdf]

Supplement

Legends of Supplemental Figures

Figure S1. Specificity verification of SUMO1 tryptic remnant antibody.

HEK293T cells were transfected with different plasmid (vector plasmid, SUMO1 plasmid or SUMO2 plasmid and their ΔGG mutants), and whole cell lysates were extracted and detected by corresponding antibodies.

Figure S2. The E1, E2, and E3s of SUMOylation are highly expressed in mouse testis.

qRT-PCR was performed to determine mRNA levels of genes in different mice tissues. *Uba2*, *Ube2i*, *Pias2* and *Pias4* were highly expressed in testis. Results are represented as mean ± SEM from triplicate experiments of three mice.

Figure S3. Endogenous SUMO1-modified sites identified by Mascot and pLink.

(A) Bar chart shows the numbers of SUMO1-modified sites identified by Mascot and pLink search engines.

(B) Venn diagram shows the overlap of high-confidence SUMO1-modified sites among three search engines.

Figure S4. Representative MS2 spectra of the corresponding SUMOylated peptides of endogenous Nab1-K479 and Eid3-K29.

The annotated MS2 spectra of the SUMO1-modified peptides “VIKTEPEDSR” (Nab1-K479) and “SLAWQHLVKQEEE” (Eid3-K29) were listed.

Figure S5. Validation of SUMO1 modification for mouse Nsrp1 and human NAB1.

(A) Mouse Nsrp1 was modified by SUMO1 at lysine 209. HEK293T cells were transfected with wild-type Nsrp1 or K209R mutant. SUMOylated bands were detected by Western blot analysis using anti-Flag or anti-HA antibodies. Black arrow represents unmodified Nsrp1 and the white represents the SUMO1-modified Nsrp1.

(B) Human NAB1 was modified by SUMO1 at lysine 480. HEK293T cells were transfected with wild-type NAB1 or K480R mutant. SUMOylated bands were detected by Western blot analysis using anti-Flag or anti-HA antibodies. Black arrow represents unmodified NAB1 and the white represents the SUMO1-modified NAB1.

Figure S6. pGL3-p300 promoter reporter is gradient induced by EGR1 expression.

Luciferase reporter assay used the *EP300* promoter as reporter construct and co-transfected with increased dose of EGR1 plasmid in HepG2 cells. Data was corrected for *Renilla* activity. * $P < 0.05$, ** $P < 0.01$, unpaired Student's *t*-test.

Figure S7. The immunohistochemistry staining of SUMO paralog in human testis.

The immunohistochemistry staining images are taken from Human Protein Atlas (http://www.proteinatlas.org). Refer to the cell morphology, the blue arrow represents spermatogonium, the

green arrow represents spermatocyte, and the red arrow represents sperm cell.

Legends of Supplemental Tables

Supplemental Table S1. Identified proteins with endogenous SUMO1-modified sites of sample EndoSUMO1-45.

A complete list of all the identified SUMO-modified proteins and sites of the sample EndoSUMO1-45 by MaxQuant search engine. The sites identified at least twice (PSM $\geq$ 2) were highlighted in orange.

Supplemental Table S2. Identified proteins with endogenous SUMO1-modified sites from mouse testis.

A complete list of all the identified SUMO1-modified proteins and sites in this study by MaxQuant search. Gene names, peptide sequences, SUMO1-modified sites, scores and other relevant information were listed. The sites identified at least twice (PSM $\geq$ 2) were highlighted in orange.

Supplemental Table S3. List of SUMO1-modified sites identified by Mascot and pLink search engines.

Sheet 1 is the combined list of SUMO1-modified sites from Mascot and pLink search engines. Sheet 2 is the list of SUMO1 sites from Mascot search engine. Sheet 3 is the list of SUMO1 sites from pLink search engine.

Supplemental Table S4. Oligonucleotide sequences of PCR primers.

A list of all the oligonucleotide sequences used in this study.

Supplemental Table S5. Detailed information of related primary antibodies

A list of the detailed information of primary antibodies used in this study including the supplier and catalog number.

Supplemental Table S6. Detailed information of all the experiments

A list of all the experiments analyzed in this study including the beads used, the instrument used and the corresponding raw file names.

Supplemental Table S7. The GO term analysis result using DAVID database

The GO term enrichment analysis result of enriched biological process, cellular component and molecular function for the high-confidence SUMO1 targets.

SUPPLEMENTAL FIGURES

Figure S1

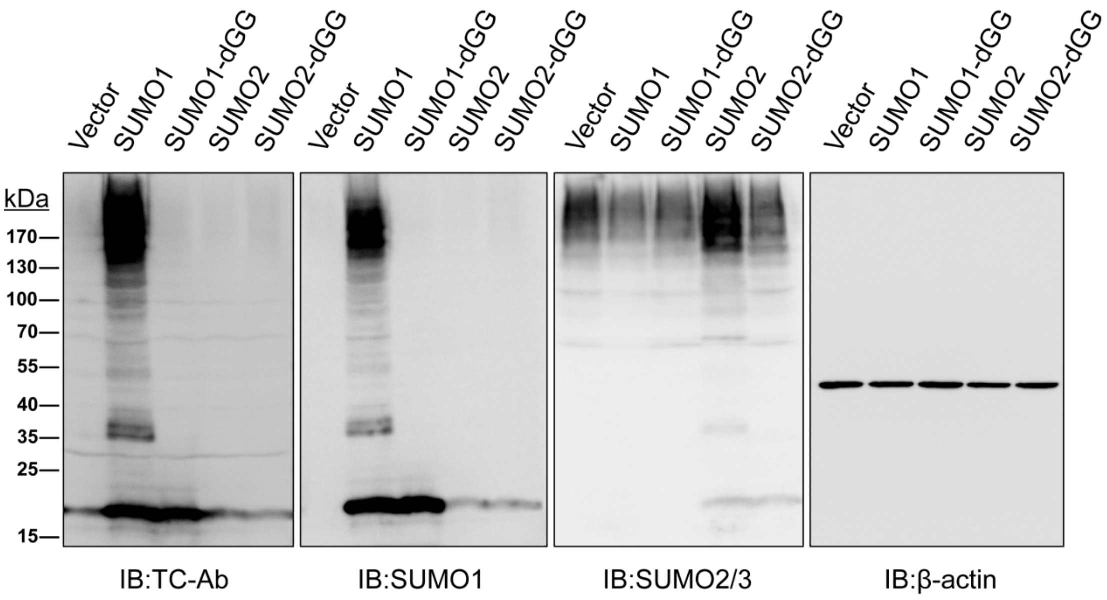

Figure S2

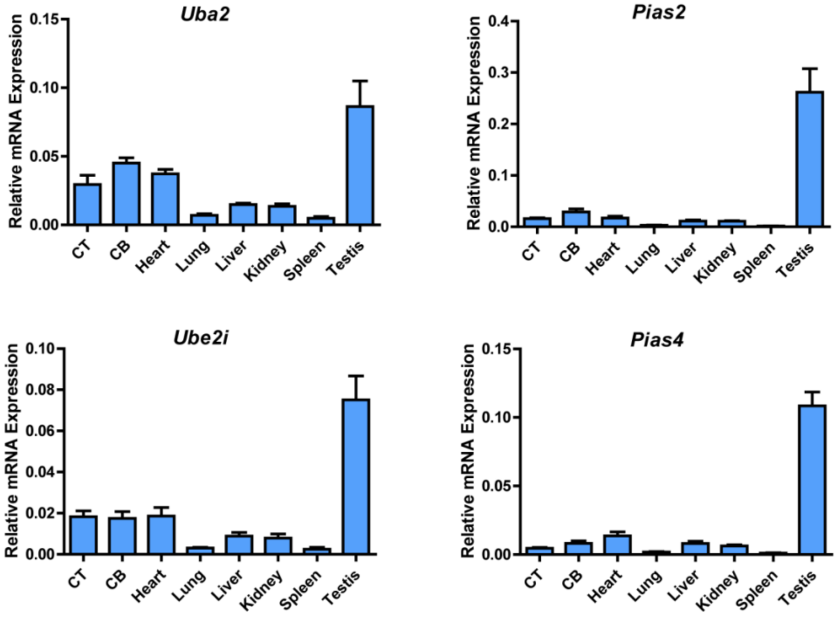

Figure S3

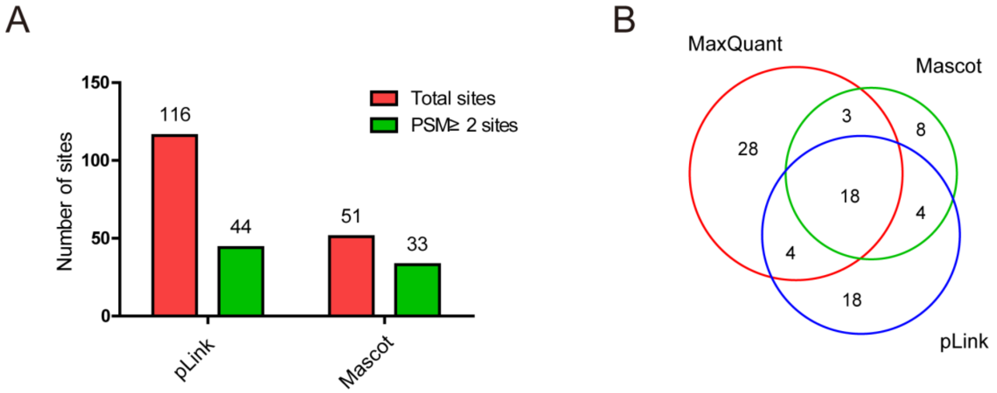
A
150
100
50
0
Number of sites
116
44
51
33
Total sites
PSM≥ 2 sites
pLink
Mascot
B
MaxQuant
Mascot
pLink
28
3
8
18
4
4
18

Figure S4

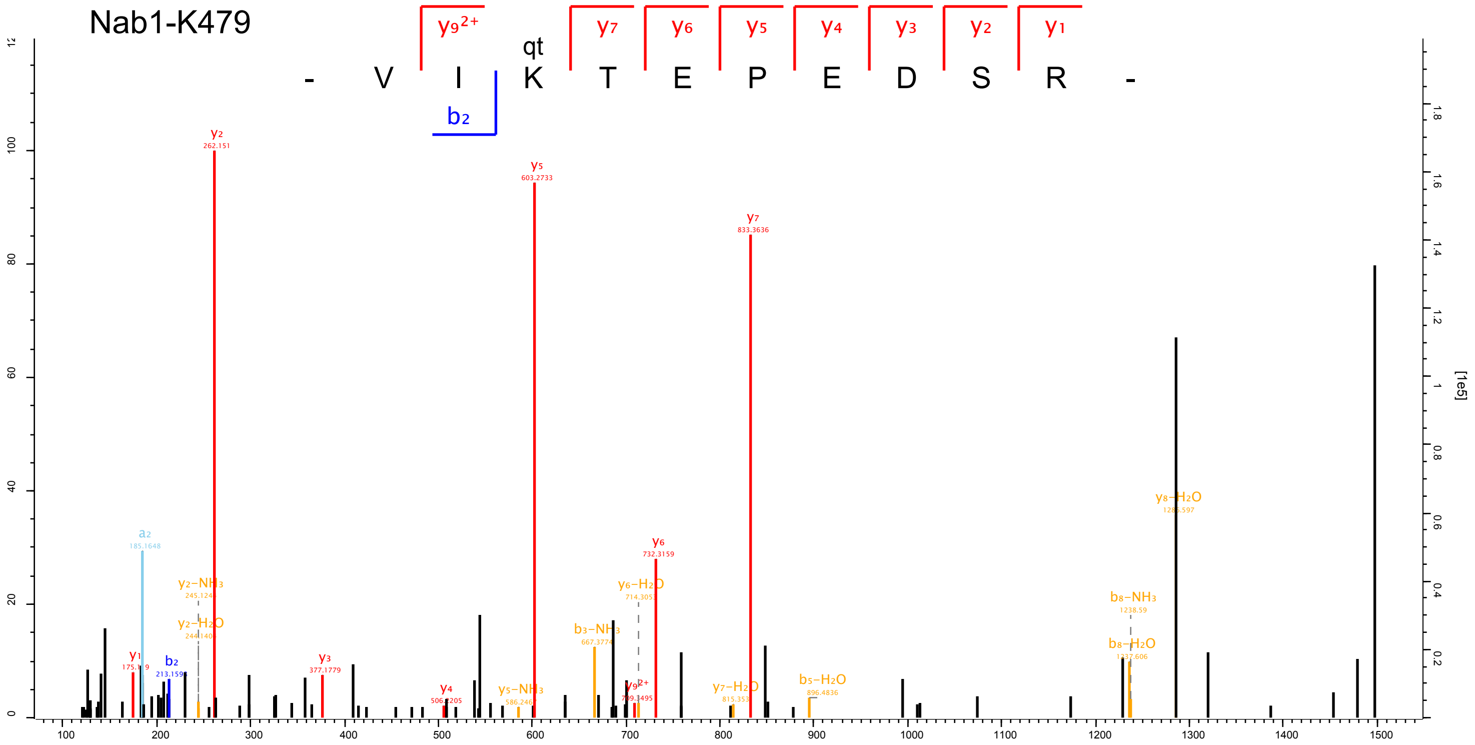

Nab1-K479
- V I K T E P E D S R -
qt
y9 2+
y7
y6
y5
y4
y3
y2
y1
b2
[1e5]

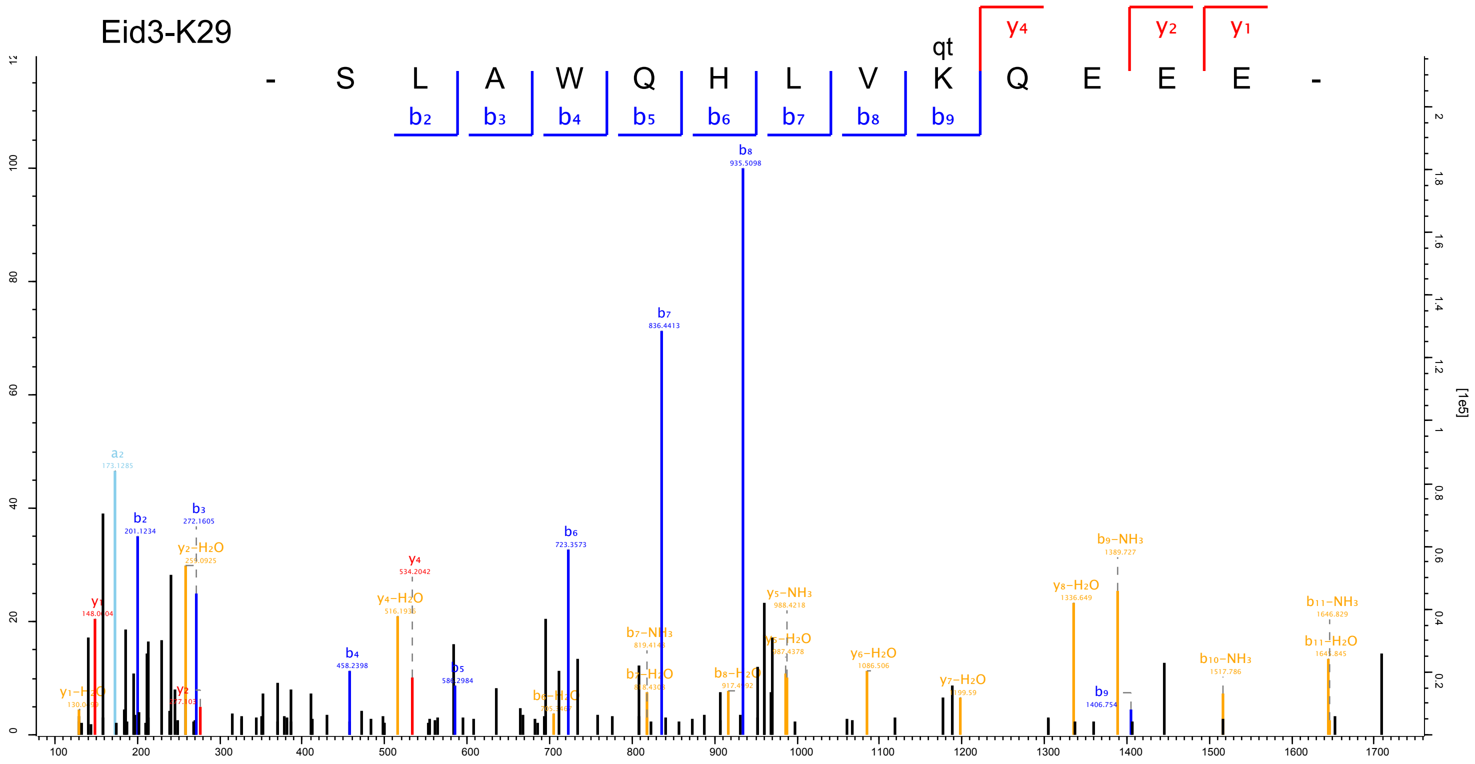

Eid3-K29
- S L A W Q H L V K Q E E E -
qt
y4
y2
y1
b2
b3
b4
b5
b6
b7
b8
b9
[1e5]

Figure S5

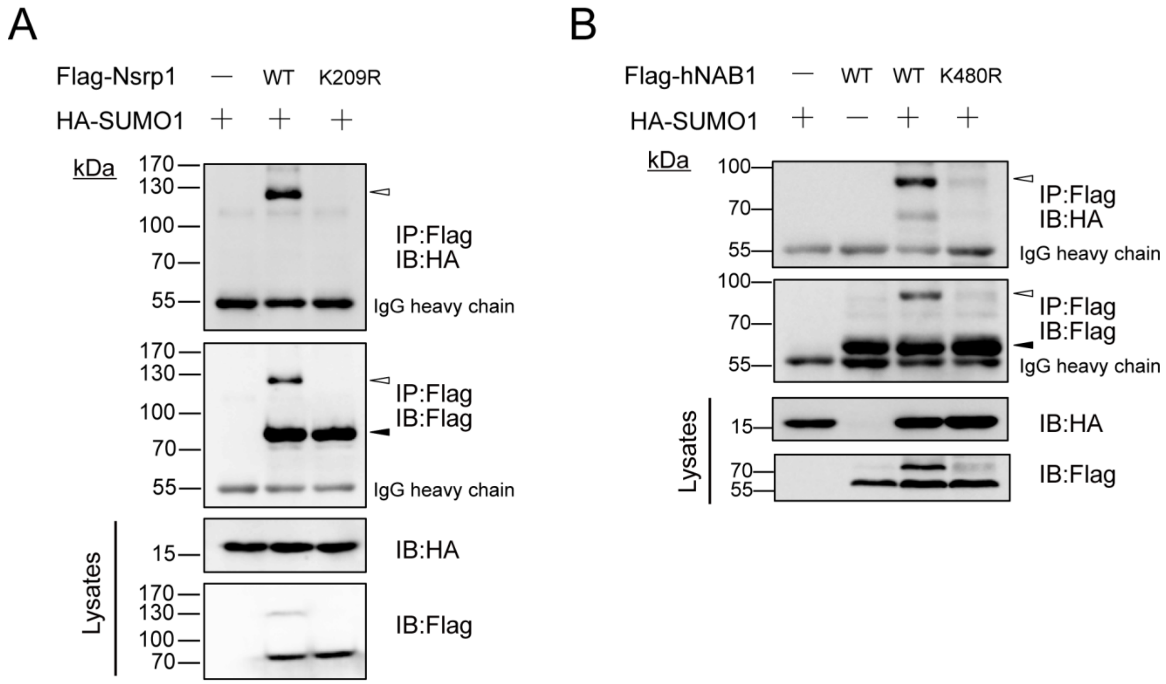
A
Flag-Nsrp1 — WT K209R
HA-SUMO1 + + +
kDa
170
130
100
70
55
IP:Flag
IB:HA
IgG heavy chain
IP:Flag
IB:Flag
IgG heavy chain
Lysates
15
IB:HA
IB:Flag
B
Flag-hNAB1 — WT WT K480R
HA-SUMO1 + — + +
kDa
100
70
55
IP:Flag
IB:HA
IgG heavy chain
IP:Flag
IB:Flag
IgG heavy chain
Lysates
15
IB:HA
IB:Flag

Figure S6

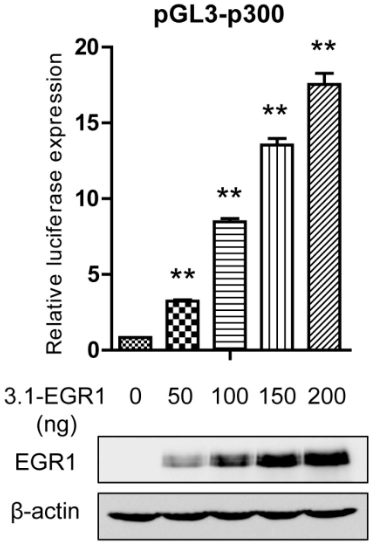
pGL3-p300
Relative luciferase expression
20
15
10
5
0
**
**
**
**
3.1-EGR1 (ng)
0
50
100
150
200
EGR1
β-actin

Figure S7

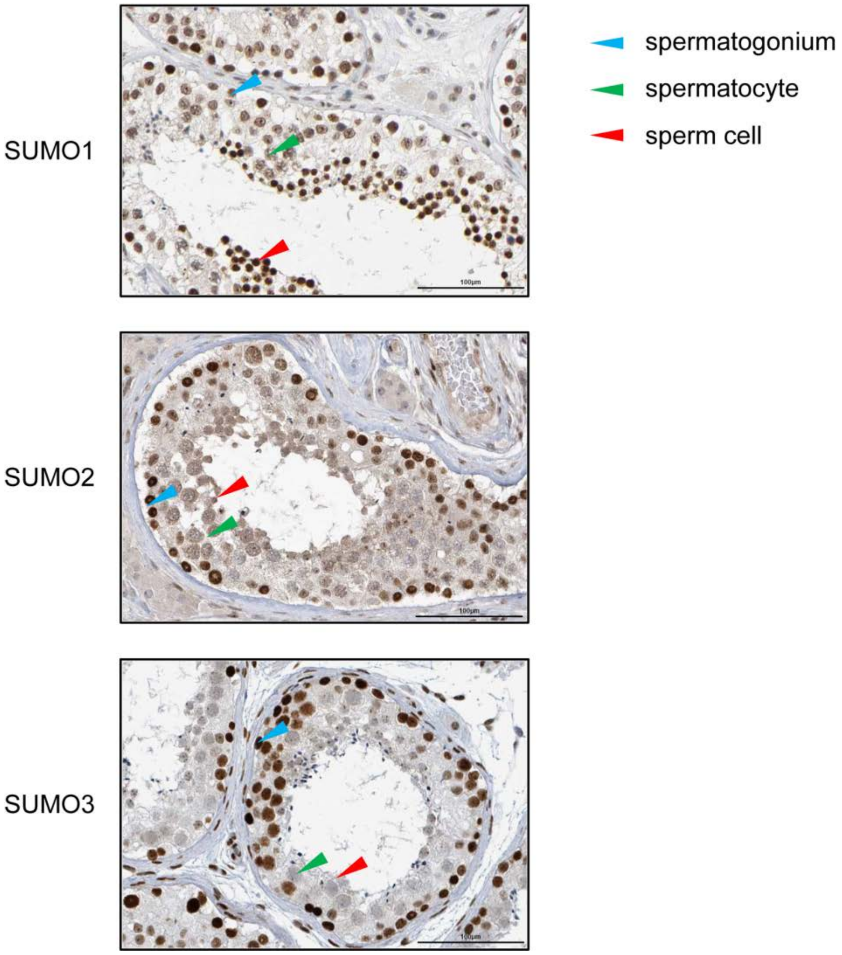

SUMO1
SUMO2
SUMO3
spermatogonium
spermatocyte
sperm cell
100μm
100μm
100μm

Supplemental Experimental Procedures

Transfection

Transfections were performed using 2 μg polyethylenimine (PEI) per 1 μg plasmid. Transfection mixtures were incubated for 20 minutes before adding to the cells. Cells were harvested after 48 hours and investigated by different approaches.

RNA isolation and real-time quantitative PCR

Mouse tissue RNA was purified using the TRIzol reagent (Invitrogen) according to the manufacturer's instructions. Total RNA was converted into cDNA by reverse transcription using PrimeScript RT reagent Kit (TaKaRa). Real-time quantitative PCR was performed using 7900HT Fast Real-Time PCR System (Applied Biosystems). The PCR reaction system was performed in a 10 μL volume containing cDNA, SYBR Green Realtime PCR Master Mix (TaKaRa) and specific primers.

Data processing for Mascot and pLink

Raw MS data files were also processed using Mascot (version 2.3.01, Matrix Science) and pLink (version 1.2) (1). As for Mascot, spectral data were searched against mouse protein RefSeq database in Proteome Discoverer 1.4.1.14 using Mascot. A target-decoy-based strategy was applied to control both peptide- and protein-level false discovery rates (FDRs) lower than 1% (2). The mass tolerance was set to be 10 ppm for precursor, and it was set 50 mmu for the tolerance of product ions. The threshold for quality control in

QTGG-modified peptides identification was ion score >20.

As for pLink, the raw MS files were converted into mgf files using pXtract software (version 2.0). The SUMO1 remnant peptide was set as a linker chain. Apart from QTGG, VYQEQTGG was used as another linker chain of SUMO1 for pLink searching. After searching, the spectra were screened using pLable software (version 2.4) for visualization. For pLink result data, QTGG-modified or VYQEQTGG-modified peptides were filtered by requiring E-value $< 1\times10\text{-}3$, and the FDR was less than 0.01, in which the FDR was calculated as described before for chemically cross-linked peptides(1).

Reference

1. Yang, B., Wu, Y. J., Zhu, M., Fan, S. B., Lin, J., Zhang, K., Li, S., Chi, H., Li, Y. X., Chen, H. F., Luo, S. K., Ding, Y. H., Wang, L. H., Hao, Z., Xiu, L. Y., Chen, S., Ye, K., He, S. M., and Dong, M. Q. (2012) Identification of cross-linked peptides from complex samples. *Nat Methods* 9, 904-906
2. Elias, J. E., and Gygi, S. P. (2007) Target-decoy search strategy for increased confidence in large-scale protein identifications by mass spectrometry. *Nat Methods* 4, 207-214

Supplemental Table S4. Oligonucleotide sequences of PCR primers

Primer name	Forward primer (5'-3')	Reverse primer (5'-3')
Primers for real-time quantitative RT-PCR		
Actb	GGCTCCTAGCACCATGAAGA	AGGGTGTAAAACGCAGCTCAG
Uba2	GCGAGTCCATTTAGCGGAGA	CTATCAAGCCGGCGATCACT
Ube2i	GGAAAGCCTGGAGGAAGGAC	GATGAAACAGTGGGGGCTCA
Pias2	GGATGGTAGCTCATCACCCG	AGGTGGGCTTAGTGTCTTGC
Pias4	CGAATTGCTGAAGCCCACTG	CAGCGATGTTGGGTGGGTAT
Primers for expression plasmids construction		
Flag-hNAB1	AGAAGCTTATGGCTGCGGCCTTACCCAG (*HindIII*)	CGTCTAGACTATCTTGAATCTTCAGGCTCTG (*XbaI*)
Flag-mNab1	AGAAGCTTATGGCCACAGCCTTACCTAGG (*HindIII*)	CGTCTAGACTATCTTGAGTCTTCAGGCTCTG (*XbaI*)
hNAB1-K480R	CTTGAATCTTCAGGCTCTGTTCTGATGACTTTCTTTTCTAAG	CTTAGAAAAGAAAGTCATCAGAACAGAGCCTGAAGATTCAAG
mNab1-K479R	TCTTCAGGCTCTGTTCTAATAACTTTCTTTTCCAAGGTG	CACCTTGGAAAAGAAAGTTATTAGAACAGAGCCTGAAGA
Flag-Nsrp1	AGAAGCTTATGGCGATCCCGGGCAGGC (*HindIII*)	GCGAATTCTTAGTCATCTTCTTTCTCAA (*EcoRI*)
Nsrp1-K209R	GTAACCCCTGAGCTTCTCTTCTCTTATCACAGTCCTGGCTTC	GAAGCCAGGACTGTGATAAGAGAAGAGAAGCTCAGGGGTTAC
Flag-Eid3	AGAAGCTTATGTCTAAAGAAAAATGTTCC (*HindIII*)	CTGAATTCTTAATATGAGTTTTTGATCATAG (*EcoRI*)
Eid3-K29R	CTCCTCCTGTCTCACCAGGTGCTGCCATG	CATGGCAGCACCTGGTGAGACAGGAGGAG
3.1-EGR1	CGGGGTACCATGGCCGCGGCCAAGGCCGAGAT (*KpnI*)	CGGAATTCTTAGCAAATTTCAATTGTCCTGGGAG (*EcoRI*)
Primers for luciferase plasmids construction		
pGL3-p300 (-1572~-44)	GCGGTACCCTCGTAACTATCCACCGACTG (*KpnI*)	GCAAGCTTCAGGAAATCTCTTCTTCGGCCCG (*HindIII*)

Supplemental Table S5. Detailed information of related primary antibodies

Primary antibody	Supplier	Cat. Number
SUMO-1 Rab MAb	Abcam	ab32058
SUMO-2/3 Rab MAb	Abcam	ab109005
HA-Tag Mouse mAb	Abmart	M20003
Flag-Tag Mouse mAb	Abmart	M20008
GFP-Tag Mouse mAb	Abmart	M20004
Anti-EGR1	Cell Signalling Technology	4153

Supplemental Table S6. Detailed information of experiments

Exp. No	Enzyme	Beads used	Instrument	Raw files
IgG_control	Trypsin+Glu-C	Agarose beads	Q-Exactive Plus	EndoSUMO1-IgG_Ctrl
EndoSUMO1-01	Lys-C+Glu-C	Agarose beads	Q-Exactive Plus	EndoSUMO1-01
EndoSUMO1-02	Trypsin+Glu-C	Agarose beads	Q-Exactive Plus	EndoSUMO1-02
EndoSUMO1-04	Lys-C+Glu-C	Agarose beads	Q-Exactive Plus	EndoSUMO1-04
EndoSUMO1-05	Trypsin+Glu-C	Agarose beads	Q-Exactive Plus	EndoSUMO1-05
EndoSUMO1-07	Trypsin+Glu-C	Agarose beads	Q-Exactive Plus	EndoSUMO1-07
EndoSUMO1-09	Trypsin+Glu-C	Agarose beads	Q-Exactive Plus	EndoSUMO1-09
EndoSUMO1-10	Trypsin+Glu-C	Agarose beads	Q-Exactive Plus	EndoSUMO1-10
EndoSUMO1-12	Trypsin+Glu-C	Agarose beads	Q-Exactive Plus	EndoSUMO1-12
EndoSUMO1-30	Trypsin+Glu-C	Agarose beads	Q-Exactive HF	EndoSUMO1-30
EndoSUMO1-31	Trypsin+Glu-C	Agarose beads	Q-Exactive HF	EndoSUMO1-31
EndoSUMO1-32	Trypsin+Glu-C	Agarose beads	Q-Exactive HF	EndoSUMO1-32
EndoSUMO1-33	Trypsin+Glu-C	Agarose beads	Q-Exactive HF	EndoSUMO1-33
EndoSUMO1-34	Trypsin+Glu-C	Agarose beads	Q-Exactive HF	EndoSUMO1-34
EndoSUMO1-35	Trypsin+Glu-C	Dynabeads	Q-Exactive HF	EndoSUMO1-35
EndoSUMO1-36	Trypsin+Glu-C	Dynabeads	Q-Exactive HF	EndoSUMO1-36
EndoSUMO1-37	Trypsin+Glu-C	Dynabeads	Q-Exactive HF	EndoSUMO1-37
EndoSUMO1-38	Trypsin+Glu-C	Dynabeads	Q-Exactive HF	two runs:<br>EndoSUMO1-38-1<br>EndoSUMO1-38-2
EndoSUMO1-39	Trypsin+Glu-C	Dynabeads	Q-Exactive HF	EndoSUMO1-39
EndoSUMO1-40	Trypsin+Glu-C	Agarose beads	Q-Exactive HF	EndoSUMO1-40
EndoSUMO1-41	Trypsin+Glu-C	Agarose beads	Q-Exactive HF	EndoSUMO1-41
EndoSUMO1-42	Trypsin+Glu-C	Agarose beads	Q-Exactive HF	EndoSUMO1-42
EndoSUMO1-43	Trypsin+Glu-C	Dynabeads	Q-Exactive HF	two runs:<br>EndoSUMO1-43-1<br>EndoSUMO1-43-2
EndoSUMO1-44	Trypsin+Glu-C	Dynabeads	Q-Exactive HF	EndoSUMO1-44
EndoSUMO1-45	Trypsin+Glu-C	Dynabeads	Q-Exactive HF	3 fractions 9 runs:<br>EndoSUMO1-45_1-1<br>EndoSUMO1-45_1-2<br>EndoSUMO1-45_1-3<br>EndoSUMO1-45_2-1<br>EndoSUMO1-45_2-2<br>EndoSUMO1-45_2-3<br>EndoSUMO1-45_3-1<br>EndoSUMO1-45_3-2<br>EndoSUMO1-45_3-3
EndoSUMO1-46	Trypsin+Glu-C	Dynabeads	Q-Exactive HF	EndoSUMO1-46
EndoSUMO1-47	Trypsin+Glu-C	Dynabeads	Q-Exactive HF	EndoSUMO1-47
